# Immunoregulatory potential of mesenchymal stem cells following activation by macrophage-derived soluble factors

**DOI:** 10.1186/s13287-019-1156-6

**Published:** 2019-02-13

**Authors:** Laura Saldaña, Fátima Bensiamar, Gema Vallés, Francisco J. Mancebo, Eduardo García-Rey, Nuria Vilaboa

**Affiliations:** 1 Hospital Universitario La Paz-IdiPAZ, Paseo de la Castellana 261, 28046 Madrid, Spain; 2 Centro de Investigación Biomédica en Red de Bioingeniería, Biomateriales y Nanomedicina, CIBER-BBN, Madrid, Spain; 3 Departamento de Cirugía Ortopédica y Traumatología, Hospital Universitario La Paz-IdiPAZ, Madrid, Spain

**Keywords:** Mesenchymal stem cells, Immunomodulation, Macrophage polarization, Cytokines, Priming, Hydrogels, Tissue repair

## Abstract

**Background:**

Immunoregulatory capacity of mesenchymal stem cells (MSC) is triggered by the inflammatory environment, which changes during tissue repair. Macrophages are essential in mediating the inflammatory response after injury and can adopt a range of functional phenotypes, exhibiting pro-inflammatory and anti-inflammatory activities. An accurate characterization of MSC activation by the inflammatory milieu is needed for improving the efficacy of regenerative therapies. In this work, we investigated the immunomodulatory functions of MSC primed with factors secreted from macrophages polarized toward a pro-inflammatory or an anti-inflammatory phenotype. We focused on the role of TNF-α and IL-10, prototypic pro-inflammatory and anti-inflammatory cytokines, respectively, as priming factors for MSC.

**Methods:**

Secretion of immunoregulatory mediators from human MSC primed with media conditioned by human macrophages polarized toward a pro-inflammatory or an anti-inflammatory phenotype was determined. Immunomodulatory potential of primed MSC on polarized macrophages was studied using indirect co-cultures. Involvement of TNF-α and IL-10 in priming MSC and of PGE_2_ in MSC-mediated immunomodulation was investigated employing neutralizing antibodies. Collagen hydrogels were used to study MSC and macrophages interactions in a more physiological environment.

**Results:**

Priming MSC with media conditioned by pro-inflammatory or anti-inflammatory macrophages enhanced their immunomodulatory potential through increased PGE_2_ secretion. We identified the pro-inflammatory cytokine TNF-α as a priming factor for MSC. Notably, the anti-inflammatory IL-10, mainly produced by pro-resolving macrophages, potentiated the priming effect of TNF-α. Collagen hydrogels acted as instructive microenvironments for MSC and macrophages functions and their crosstalk. Culturing macrophages on hydrogels stimulated anti-inflammatory versus pro-inflammatory cytokine secretion. Encapsulation of MSC within hydrogels increased PGE_2_ secretion and potentiated immunomodulation on macrophages, attenuating macrophage pro-inflammatory state and sustaining anti-inflammatory activation. Priming with inflammatory factors conferred to MSC loaded in hydrogels greater immunomodulatory potential, promoting anti-inflammatory activity of macrophages.

**Conclusions:**

Factors secreted by pro-inflammatory and anti-inflammatory macrophages activated the immunomodulatory potential of MSC. This was partially attributed to the priming effect of TNF-α and IL-10. Immunoregulatory functions of primed MSC were enhanced after encapsulation in hydrogels. These findings may provide insight into novel strategies to enhance MSC immunoregulatory potency.

**Electronic supplementary material:**

The online version of this article (10.1186/s13287-019-1156-6) contains supplementary material, which is available to authorized users.

## Background

The inflammatory response to tissue injury is essential for the correct restoration of tissue structure and function. However, an uncontrolled or unresolved inflammatory process can lead to chronic inflammation and further tissue damage. Macrophages are key regulators of wound healing and are involved in both advancing and resolving inflammation by secreting multiple cytokines and growth factors. Macrophages exhibit functional transitions as tissue repair progresses and can adopt a wide spectrum of phenotypes. Two of the best-characterized phenotypes are pro-inflammatory or M1-like phenotype and anti-inflammatory or M2-like phenotype. M1 macrophages produce high levels of pro-inflammatory cytokines and are related to the early stage of inflammation whereas M2 macrophages, with lower pro-inflammatory cytokine production, are associated with the resolution of inflammation and tissue repair [1]. There is evidence that macrophages can display more complex phenotypes with traits associated with both M1 and M2 activation states [2, 3]. In addition, mixed populations of macrophages have been identified [4, 5]. Functional repolarization of macrophages toward an anti-inflammatory phenotype ensures proper return to homeostasis after injury and is mediated by a large panel of mediators including prostaglandin E_2_ (PGE_2_) [6]. Several studies suggest that an incorrect balance between M1- and M2-like activities after injury can lead to persistent inflammation and/or maladaptive repair processes, both contributing to aberrant tissue repair [7, 8]. Due to their critical role during wound healing, macrophages have emerged as potential targets in therapeutic tissue regeneration strategies [9].

Accumulating evidence suggests that mesenchymal stem cells (MSC) promote tissue repair and regeneration through modulation of immune response and secretion of growth factors rather than by replacement of damaged cells. MSC release a wide range of immunoregulatory factors including PGE_2_ and interleukin-6 (IL-6) that skew macrophages toward a pro-resolving profile [10]. Immunoregulatory capacity of MSC is not constitutive, but depends on a process of “licensing” that implies the activation of MSC by the inflammatory milieu. Thus, in response to inflammatory mediators, MSC produce soluble factors that regulate the immune response. The requirement of MSC activation to induce immunoregulation is supported by data showing that suppression of T cells proliferation induced by MSC in co-cultures was only achieved after addition of sufficient levels of interferon-γ (IFN-γ) and tumor necrosis factor-α (TNF-α) [11–13]. Macrophages plasticity leads to changes in the balance between pro-inflammatory and anti-inflammatory factors as tissue is healed and remodeled. The earliest events trigger the release of numerous pro-inflammatory mediators, which are followed by a shift to increased production of anti-inflammatory cytokines and growth factors to allow tissue repair [14]. Additionally, pro-inflammatory and anti-inflammatory cytokine expression can be induced simultaneously at early stages of inflammation [15]. Given the variability of macrophage activation states throughout the course of inflammation and tissue repair, it is expected that MSC establish interactions with different macrophage phenotypes and that both pro-inflammatory and anti-inflammatory cytokines influence MSC-mediated immunomodulation. To date, the effects of the cocktail of factors originated from pro-inflammatory or anti-inflammatory macrophage populations on immunomodulatory properties of MSC have not been described.

MSC, like all somatic tissues, express human leukocyte antigens (HLA) class I constitutively and have the ability to express HLA class II when exposed to inflammatory factors. The HLA class I antigens are associated with the activation of CD8+ T lymphocytes while HLA class II antigens are recognized by CD4+ T lymphocytes. MSC appear to evade immune rejection by modulating T cell phenotype and immunosuppressing the local environment. A number of clinical trials involving allogeneic MSC transplantation have shown overall safety and potential effectiveness [16]. MSC have been employed in the clinical treatment of graft-versus-host disease (GvHD) due to their ability to inhibit proliferation and cytotoxic activity of immune system cells. A limited number of clinical trials have reported humoral alloimmunization in human subjects receiving mismatched MSC, but it remains unclear whether this has an impact on their therapeutic efficacy [17]. There is growing interest in combining MSC with hydrogels prepared with extracellular matrix (ECM) proteins that resemble the microenvironments where they reside in order to prolong cell survival, potentiate their function, and prevent rejection by the host [18, 19]. In this work, we extensively investigated the immunomodulatory functions of human MSC activated with secreted factors from human monocyte-derived macrophages polarized toward a pro-inflammatory or an anti-inflammatory phenotype using standard two-dimensional (2D) culture conditions. We focused on the role of TNF-α and IL-10, prototypic pro-inflammatory and anti-inflammatory cytokines, respectively, as priming factors for MSC. Immunoregulatory potential of MSC was evaluated in co-cultures with pro-inflammatory or anti-inflammatory macrophage populations. The assays that led to the most informative data were then performed using MSC encapsulated in collagen hydrogels, which represent a more physiological relevant model.

## Methods

### Isolation and culture of primary human macrophages

**Fig. 1:**
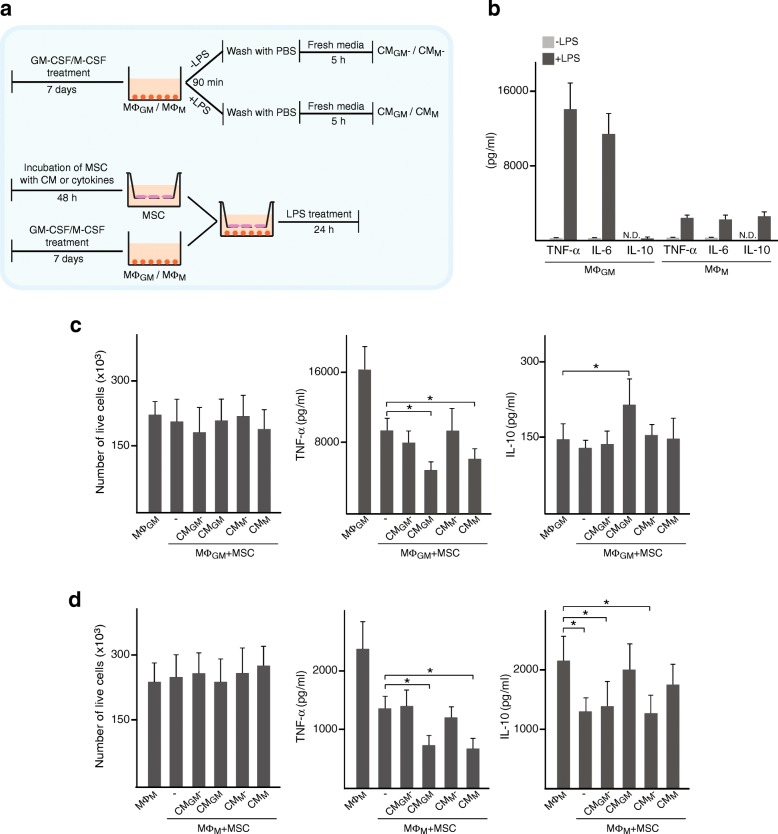
Immunomodulatory effects of MSC primed with CM from macrophages. **a** Scheme used to generate conditioned media (CM) from macrophages (upper row). MΦ_GM_ or MΦ_M_ were treated (CM_GM_ or CM_M,_ respectively) or not (CM_GM_− or CM_M_−, respectively) with LPS for 90 min, thoroughly washed with PBS to remove LPS, and incubated in fresh media for 5 h. Scheme of the set-up of co-cultures (lower row). MSC were incubated or not with CM from macrophages or with cytokines for 48 h, thoroughly washed with PBS, and co-cultured with MΦ_GM_ or MΦ_M_ in the presence of LPS for 24 h. **b** Levels of inflammatory cytokines in CM of MΦ_GM_ or MΦ_M_ stimulated or not with LPS. Number of MΦ_GM_ (**c**) or MΦ_M_ (**d**) cultured in isolation or co-cultured with MSC primed or not (−) with CM (left graphs) and levels of TNF-α (middle graphs) and IL-10 (right graphs) in media. *p < 0.05. N.D., not detected

Buffy coats were obtained from 30 healthy blood donors, as anonymously provided by the Comunidad de Madrid Blood Bank (Madrid, Spain). Ethical approvals for all blood sources and processes used in this study were approved by the Human Research Committee of Hospital Universitario La Paz (Date of Approval: 03/06/2015). All experiments were carried out in accordance with the approved guidelines and regulations. Human peripheral blood mononuclear cells (PBMC) were isolated from buffy coats by density gradient centrifugation using Ficoll-Paque Plus (GE Healthcare Bio-sciences, Uppsala, Sweden). For monocyte isolation, PBMC were seeded at a density of 15 × 10^6^/well in six-well plates and allowed to adhere for 1 h in serum-free RPMI (Lonza, Basel, Switzerland). Adherent cells were cultured for 7 days in RPMI supplemented with 10% (*v*/*v*) heat-inactivated fetal bovine serum (FBS) and 200 U/ml of granulocyte macrophage-colony stimulating factor (GM-CSF) or 20 ng/ml macrophage-colony stimulating factor (M-CSF) (both from Peprotech, London, UK). Cytokines were added every 2 days. Macrophages generated in the presence of GM-CSF or M-CSF are referred to as MΦ_GM_ and MΦ_M_, respectively. Conditioned media (CM) were obtained from MΦ_GM_ or MΦ_M_ that were treated or not with 10 ng/ml lipopolysaccharide (LPS) (Sigma, Madrid, Spain) for 90 min, washed three times with phosphate-buffered saline (PBS), and cultured in RPMI medium supplemented with 10% FBS for 5 h. The CM were clarified by centrifugation at 1200*g* for 10 min. The experimental scheme used to generate CM is shown in Fig. 1a.

### MSC culture and co-culture with macrophages

Purified human bone marrow-derived MSC were purchased from Lonza and expanded in a defined medium (Lonza) consisting of basal medium and supplement mix. All experiments were performed between passages 5 and 7 using cells isolated from six different donors aged 18–30 years. 10^5^ MSC were seeded in the upper chamber of a 24-mm-diameter transwell insert with 0.4-μm pores (Corning, Lowell, MA, USA) and incubated for 48 h in 3 ml of DMEM supplemented with 15% (*v*/*v*) heat-inactivated FBS or in 3 ml of a mixture of equal volumes of DMEM with 15% FBS and CM from macrophages. When indicated and prior to addition to MSC, CM were incubated for 1 h at 37 °C with 1 μg/ml neutralizing antibody against TNF-α or IL-10 (Biolegend, San Diego, CA, USA). Parallel sets of MSC were treated for 48 h with 1 or 10 ng/ml TNF-α, 0.1 or 1 ng/ml IL-10, or combinations of both cytokines (Peprotech). These doses were selected based on the concentrations of TNF-α and IL-10 in the mixtures of DMEM and CM from LPS-stimulated MΦ_GM_ or MΦ_M_ used for MSC treatments. MSC treated with CM or cytokines are referred to as primed MSC. The transwells with unprimed or primed MSC were washed with PBS and transferred to six-well plates containing cultures of MΦ_GM_ or MΦ_M_ and incubated for 24 h in 3 ml of a mixture of equal volumes of RPMI and DMEM containing 12.5% FBS and 10 ng/ml LPS. When indicated, 1 μg/ml antibody against PGE_2_ (Cayman Chemical Company, Ann Arbor, MI, USA) or IL-6 (R&D Systems, Wiesbaden, Germany) was added along with LPS. At the end of the incubation period, the number of live macrophages was determined by the trypan blue dye exclusion test. The experimental scheme used for setting co-cultures is shown in Fig. 1a. In some experiments, 10^5^ MSC were seeded in 12-well plates and incubated with CM or cytokines as described above. After 48 h, MSC were washed with PBS and further incubated for 24 h in fresh culture media, as shown in the experimental scheme in Fig. 4a. To assess that MSC modulate cytokine secretion of stimulated macrophages in the absence of LPS, macrophages were treated with LPS for 90 min, washed with PBS, and then co-cultured with MSC for 5 h in fresh media (see experimental scheme in Additional file 1: Figure S2).

### Collagen gel co-cultures

Hydrogels (HG) containing 1.5 mg/ml collagen were prepared by mixing at 4 °C 40 μl of 10X DMEM, 10 μl of 1 N NaOH, 162 μl of H_2_O, 8 μl of 7.5% NaHCO_3_, 100 μl of serum-free DMEM, and 180 μl of 5 mg/ml rat-tail type I collagen diluted in 0.1 M acetic acid (Ibidi GmbH, Martinsried, Germany). 10^5^ MSC, previously treated or not for 48 h with CM, were resuspended in 100 μl of serum-free DMEM and added to the solution. HG-lacking cells were used as controls. After homogenizing the mixture by pipetting, 600 μl of suspension were distributed per well of 24-well plates and incubated at 37 °C for 30 min. After polymerization, 600 μl of RPMI supplemented with 25% (*v*/*v*) FBS were added and 2 × 10^5^ MΦ_GM_ or MΦ_M_ were seeded onto HG loaded or not with MSC. Then, HG media were supplemented with 10 ng/ml LPS and incubated for 24 h. For comparative purposes, macrophages were seeded on 24-well plates made of tissue culture plastic (TCP) and incubated for 24 h in 1200 μl of a mixture of equal volumes of RPMI and DMEM containing 12.5% FBS and 10 ng/ml LPS. In the case of MSC, cells were seeded on TCP or encapsulated in HG and further incubated for 24 h in the same media without LPS. The experimental scheme used is shown in Fig. 8a. The cell morphology was observed under a phase-contrast microscope (Nikon Diaphot, Tokio, Japan).

### Flow cytometry assays

Immunofluorescence staining of cell surface antigens in MSC was performed by incubating cells for 30 min at 4 °C in the dark with mouse anti-human leukocyte antigen (HLA)-DR, DP, DQ (HLA class II)-FITC, HLA-ABC (HLA class I)-APC, CD34-FITC, CD44-FITC, CD105-PE, CD29-APC, and CD45-APC Abs (all from BD Biosciences, San Jose, CA,USA). Phenotypic characterization of macrophages generated by incubation with GM-CSF or M-CSF was assessed by staining with CD163-PE, CD197 (CCR7)-FITC, and CD80-APC (all from Miltenyi Biotec, Bergisch-Gladbach, Germany). Cells incubated in the absence of antibodies were used as controls. After incubation, cells were washed three times with PBS, fixed with 1% (*w*/*v*) formaldehyde in PBS, and analyzed by flow cytometry using a FACSCalibur analyzer and CellQuest software (both from BD Biosciences).

### Immunoenzymatic assays

The culture media were clarified by centrifugation at 1200*g* for 10 min; supplemented with 2 μg/ml aprotinin, 17.5 μg/ml phenyl-methylsulfonyl fluoride, 1 μg/ml pepstatin A, and 50 μg/ml bacitracin (Sigma); and stored at − 80 °C. Levels of TNF-α, IL-10, and IL-6 in cell culture media were determined using BD CBA Flex Sets (BD Biosciences). The data were acquired using a FACSCalibur flow cytometer and analyzed with the FCAP Array Software version 3.0 (BD Biosciences). The detection limits of the CBA Flex Sets were 3.7 pg/ml for TNF-α, 2.5 pg/ml for IL-6, and 3.3 pg/ml for IL-10. PGE_2_ levels were measured using a human-specific ELISA kit (Cayman) with a detection limit of 15 pg/ml.

### Gene expression

Total RNA was isolated using TRI Reagent (Molecular Research Center, Inc., Cincinnati, OH, USA). Complementary DNAs were prepared from total RNA using the Transcriptor Reverse Transcriptase and an anchored-oligo (dT)_18_ primer (Roche Applied Science, Indianapolis, IN, USA). Real-time quantitative PCR was performed using LightCycler FastStart DNA Master SYBR Green I and LightCycler detector (Roche). Quantitative expression values were extrapolated from standard curves and were normalized to glyceraldehyde 3-phosphate dehydrogenase (GAPDH) values. Specific oligonucleotide primers were IL-6, 5′-CCCCAGGAGAAGATTCCAAA-3′ (forward primer, F), 5′-CCAGTGATGATTTTCACCAGG-3′ (reverse primer, R); cyclooxygenase-2 (COX-2), 5′-TGAGCATCTACGGTTTGCTG-3′ (F), 5′-TGCTTGTCTGGAACAACTGC-3′ (R); and GAPDH, 5′-GTGAAGGTCGGAGTCAACG-3′ (F), 5′-GAAGATGGTGATGGGATTTCC-3′ (R).

### Statistical analysis

The statistical analyses were performed using the Statistical Program for Social Sciences version 11.5 (SPSS Inc., Chicago, IL, USA). Data are presented as means ± SD of six independent experiments. Quantitative data were tested using two-sided Kruskal-Wallis and Mann-Whitney *U* rank-sum tests. Post hoc comparisons were analyzed by the Mann-Whitney *U* test, adjusting the *p* value with the Bonferroni correction, and the level of significance was set to *p* < 0.05.

## Results

### Priming MSC with factors secreted by pro-inflammatory or anti-inflammatory macrophages enhances their immunomodulatory potential

We primed MSC with CM from MΦ_GM_ or MΦ_M_ stimulated or not with LPS to examine the influence of inflammatory cytokines on MSC immunomodulatory potential (Fig. 1a). MΦ_GM_ expressed the M1 markers CD80 and CCR7 whereas they were devoid of cell surface CD163, a marker of M2 macrophages. In contrast, MΦ_M_ expressed high levels of CD163 and very low levels of CD80 and CCR7 (Additional file 1: Figure S1). The concentrations of inflammatory cytokines in the CM from MΦ_GM_ or MΦ_M_ correlated with their pro-inflammatory or anti-inflammatory phenotype, respectively (Fig. 1b). CM from LPS-stimulated MΦ_GM_ (CM_GM_) contained higher levels of TNF-α and IL-6 and lower levels of IL-10 than CM from LPS-stimulated MΦ_M_ (CM_M_). IL-10 levels could not be detected in CM from unstimulated macrophages, which contained low concentrations of TNF-α and IL-6 (Fig. 1b). To evaluate their immunomodulatory potential, MSC primed or not with CM were co-cultured with MΦ_GM_ or MΦ_M_ as shown in Fig. 1a. MSC did not affect macrophage viability, as numbers of live MΦ_GM_ or MΦ_M_ cultured in isolation or co-cultured with primed or unprimed MSC were similar (Fig. 1c, d, left panels). Co-culture of MΦ_GM_ with unprimed MSC decreased TNF-α levels, an effect also observed in co-cultures of MΦ_M_ (Fig. 1c, d, middle panels). MSC primed with CM from unstimulated macrophages had no effect on TNF-α secretion from MΦ_GM_ or MΦ_M_ (Fig. 1c, d, middle panels). However, MSC primed with CM from LPS-stimulated macrophages further decreased TNF-α levels in co-cultures and no differences were found between priming with CM_GM_ or CM_M_ (Fig. 1c, d, middle panels). The low IL-10 levels secreted by MΦ_GM_ were not altered when co-cultured with unprimed MSC or CM_M_-primed MSC but increased in co-cultures with CM_GM_-primed MSC (Fig. 1c, right panels). IL-10 production by MΦ_M_ was notably reduced in co-cultures with unprimed MSC (Fig. 1d, right panels). However, this reduction was not observed when MSC were primed with CM_GM_ or CM_M_. As observed for TNF-α, IL-10 levels in co-cultures were unaffected by priming MSC with CM from unstimulated macrophages (Fig. 1c, d, right panels). To assess that MSC can modulate TNF-α and IL-10 secretion of stimulated macrophages in the absence of LPS, macrophages were treated with LPS, washed, and then co-cultured with MSC in fresh media (Additional file 1: Figure S2). Under these conditions, MSC decreased TNF-α levels in co-cultures with MΦ_GM_ or MΦ_M_ and priming MSC with CM_GM_ or CM_M_ increased their immunomodulatory properties, as observed in co-cultures treated with LPS. Also, changes in IL-10 levels induced by MSC were similar in co-cultures with or without LPS. Taken together, our data show that MSC primed with CM from LPS-stimulated macrophages, which contain high levels of inflammatory mediators, display greater immunomodulatory potential than unprimed MSC.

### TNF-α and IL-10 in CM from macrophages are involved in priming MSC

**Fig. 2:**
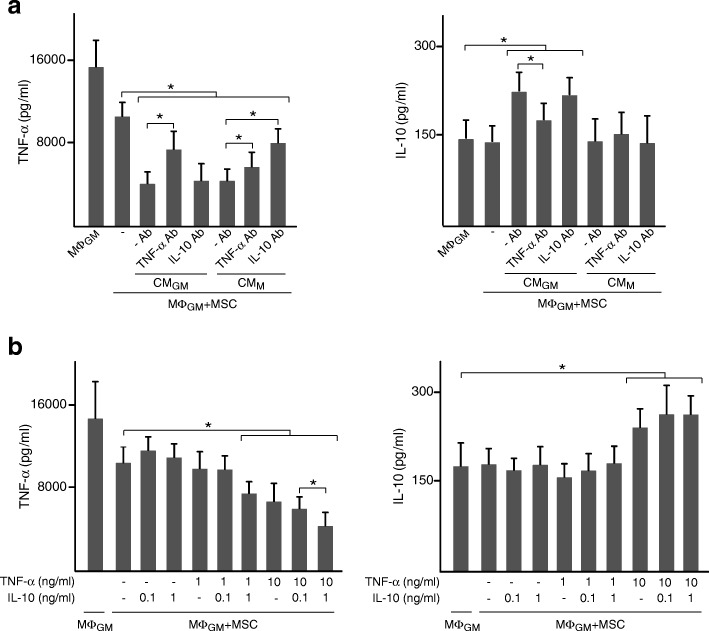
TNF-α and IL-10 prime MSC to regulate cytokine secretion from pro-inflammatory macrophages. **a** TNF-α and IL-10 levels in media of MΦ_GM_ cultured in isolation or co-cultured with MSC primed or not (−) with CM_GM_ or CM_M_. CM were incubated or not (−Ab) with neutralizing antibody (Ab) against TNF-α or IL-10. **b** TNF-α and IL-10 levels in media of MΦ_GM_ cultured in isolation or co-cultured with MSC primed or not (−) with the indicated doses of TNF-α, IL-10 or combinations of both cytokines. *p < 0.05

**Fig. 3:**
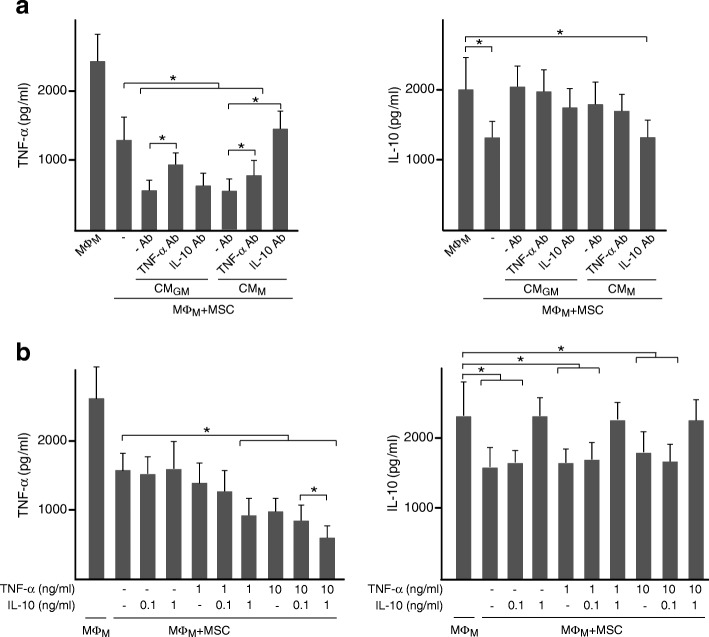
TNF-α and IL-10 prime MSC to regulate cytokine secretion from anti-inflammatory macrophages. **a** TNF-α and IL-10 levels in media of MΦ_M_ cultured in isolation or co-cultured with MSC primed or not (−) with CM_GM_ or CM_M_. CM were incubated or not (−Ab) with neutralizing antibody (Ab) against TNF-α or IL-10. **b** TNF-α and IL-10 levels in media of MΦ_M_ cultured in isolation or co-cultured with MSC primed or not (−) with the indicated doses of TNF-α, IL-10 or combinations of both cytokines. *p < 0.05

We next investigated the role of the pro-inflammatory TNF-α and the anti-inflammatory IL-10 cytokines as priming factors for MSC. For this purpose, CM from macrophages were incubated with neutralizing TNF-α or IL-10 antibody before being added to MSC. Treatment of CM_GM_ or CM_M_ with anti-TNF-α reduced the ability of primed MSC to decrease TNF-α levels in co-cultures of MΦ_GM_ (Fig. 2a, left panel). Interestingly, a modulatory effect on TNF-α secretion was also observed when CM_M_, which contained high IL-10 amounts, were treated with anti-IL-10. IL-10 secretion induced by CM_GM_-primed MSC in co-cultures of MΦ_GM_ was attenuated when CM were incubated with anti-TNF-α (Fig. 2a, right panel). Neutralization of IL-10 in CM_GM_ had no effect on TNF-α and IL-10 levels (Fig. 2a). To further investigate the effect of TNF-α and IL-10 on MSC, cells were incubated with these cytokines before co-culturing with MΦ_GM_ (Fig. 2b). IL-10 at 0.1 ng/ml had no effect on MSC immunomodulation. TNF-α levels in co-cultures were also unaffected by priming MSC with either IL-10 or TNF-α at 1 ng/ml, but decreased after incubation with both cytokines (Fig. 2b, left panel). Priming MSC with 10 ng/ml TNF-α diminished TNF-α levels in co-cultures, which further decreased when MSC were primed with 10 ng/ml TNF-α plus 1 ng/ml IL-10. Finally, IL-10 levels in co-cultures increased only when MSC were primed with 10 ng/ml TNF-α, alone or in combination with IL-10 (Fig. 2b, right panel). Regarding co-cultures of MΦ_M_, neutralization of TNF-α in CM_GM_ or CM_M_ reduced the ability of primed MSC to modulate TNF-α levels without affecting IL-10 (Fig. 3a). Moreover, blocking IL-10 in CM_M_ suppressed the regulatory effects of primed MSC. Priming effects of TNF-α on MSC in co-cultures of MΦ_M_ increased when 1 ng/ml IL-10 was added (Fig. 3b, left panel). Notably, IL-10 levels increased when MΦ_M_ were co-cultured with MSC primed with 1 ng/ml of IL-10 independently of the presence of TNF-α (Fig. 3b, right panel). Overall, these data indicate that TNF-α and IL-10 secreted from macrophages prime MSC to enhance their immunomodulatory potential.

### Immunomodulatory effects of primed MSC on macrophages are mediated by PGE_2_

**Fig. 4:**
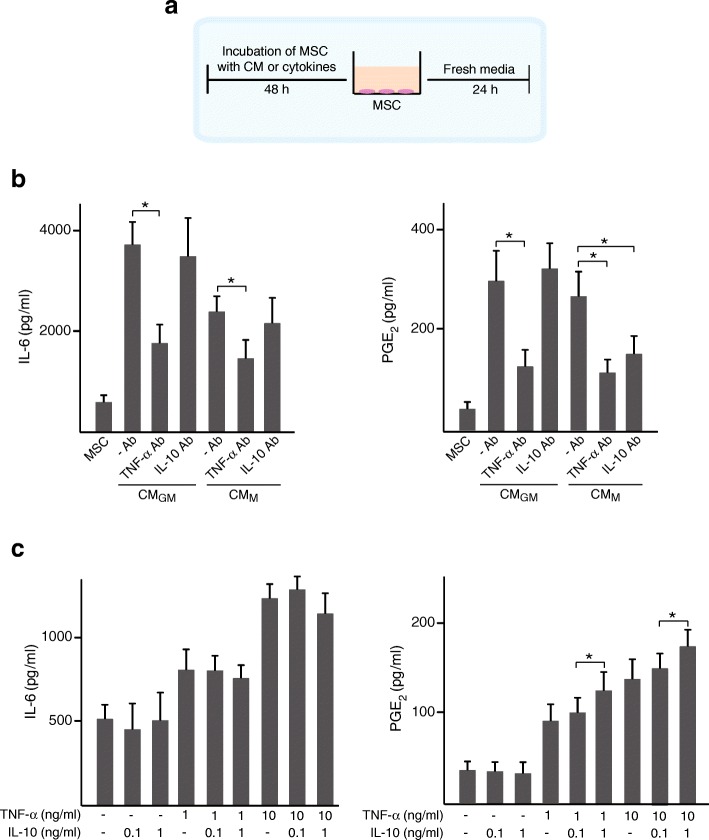
IL-6 and PGE_2_ secretion by primed MSC. **a** Scheme of MSC treatment with CM or cytokines. **b** IL-6 and PGE_2_ levels in media of MSC primed or not (−) with CM_GM_ or CM_M_. CM were incubated or not (−Ab) with neutralizing antibody (Ab) against TNF-α or IL-10. **c** IL-6 and PGE_2_ levels in media of MSC primed or not with the indicated doses of TNF-α, IL-10 or combinations of both cytokines. *p < 0.05

**Fig. 5:**
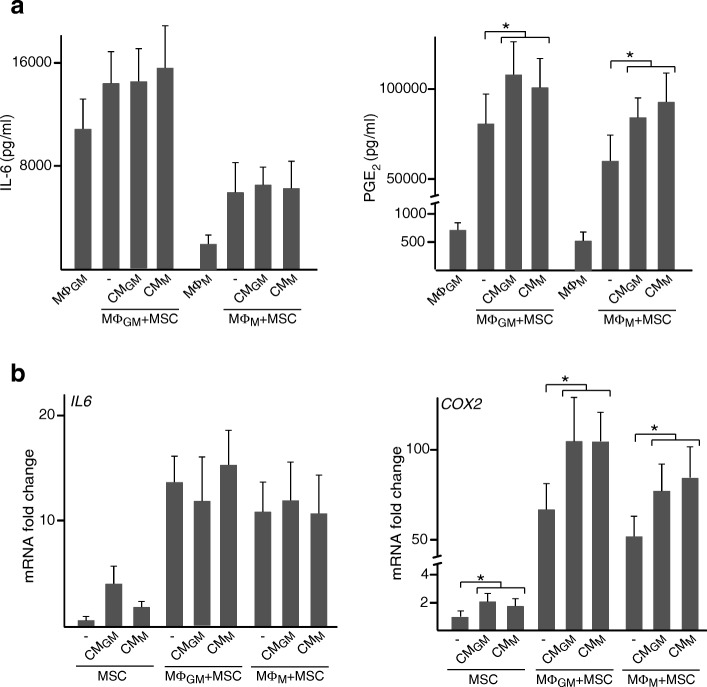
IL-6 and PGE_2_ secretion and mRNA levels in co-cultures of macrophages and primed MSC. **a** IL-6 and PGE_2_ levels in media of MΦ_GM_ or MΦ_M_ cultured in isolation or co-cultured with MSC primed or not (−) with CM_GM_ or CM_M_. **b** *IL6* and *COX2* mRNA fold changes in MSC primed or not with CM and cultured in isolation or co-cultured with MΦ_GM_ or MΦ_M._ mRNA data are relative to those measured in unprimed MSC cultured in isolation, which were given the arbitrary value of 1. *p < 0.05

Next, we examined whether the soluble mediators PGE_2_ and IL-6 are involved in the immunomodulation mediated by primed MSC. Priming MSC with CM resulted in increased secretion of IL-6, which reached higher levels after incubation with CM_GM_ than with CM_M_ (Fig. 4b, left panel). To explore whether TNF-α and IL-10 originated from macrophages play a role in this regulation, CM were incubated with neutralizing antibodies. The increase in IL-6 secretion induced by CM_GM_ or CM_M_ was attenuated by blocking TNF-α but not IL-10. Interestingly, PGE_2_ production increased to a similar extent in MSC primed with CM_GM_ or CM_M_ and this effect was largely attenuated by neutralizing TNF-α (Fig. 4b, right panel). Blocking IL-10 decreased PGE_2_ levels secreted by MSC primed with CM_M_ but not with CM_GM_. Incubation of MSC with TNF-α but not IL-10 induced a dose-dependent increase in IL-6 and PGE_2_ secretion (Fig. 4c). Notably, MSC primed with TNF-α in combination with high doses of IL-10 secreted higher PGE_2_ levels than MSC treated with TNF-α alone (Fig. 4c, right panel). This effect was not observed for IL-6 secretion (Fig. 4c, left panel). IL-6 levels were substantially higher in single-cultured MΦ_GM_ than in MΦ_M_ whereas PGE_2_ secretion was similar for both macrophage phenotypes (Fig. 5a). Co-culturing with primed or unprimed MSC led to a similar increase in IL-6 levels (Fig. 5a, left panel). PGE_2_ levels also increased upon co-culturing MΦ_GM_ or MΦ_M_ with MSC although increased further when MSC were primed with CM (Fig. 5a, right panel). No differences were found between priming with CM_GM_ or CM_M_. Next, mRNA levels of *IL6* and *COX2*, a key enzyme in PGE_2_ synthesis, were determined in MSC cultured in isolation or co-cultured with macrophages. *IL6* and *COX2* mRNA levels in single-cultured MSC correlated with IL-6 and PGE_2_ secretion profiles (Figs. 5b and 4b). *IL6* mRNA levels increased after priming MSC with CM, but to a higher extent with CM_GM_ than with CM_M_. In contrast, *COX2* transcript levels increased to the same extent after exposure to CM_GM_ or CM_M_ (Fig. 5b). *IL6* and *COX2* mRNA levels in MSC substantially increased when co-cultured with macrophages. Similar to that observed at the secretion level, *COX2* mRNA levels in primed MSC co-cultured with macrophages were higher than those in unprimed counterparts whereas these differences were not found in *IL6* transcript (Fig. 5b). These results indicate that priming with CM may potentiate the secretion of PGE_2_ from MSC in co-cultures but not of IL-6.

**Fig. 6:**
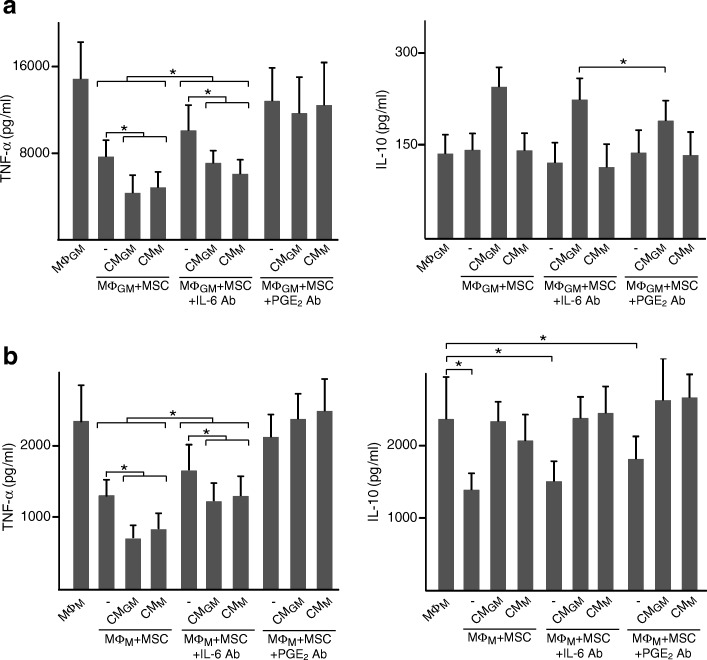
Involvement of IL-6 and PGE_2_ in the regulation of macrophage cytokine secretion by primed MSC. TNF-α and IL-10 levels in media of MΦ_GM_ (**a**) or MΦ_M_ (**b**) cultured in isolation or co-cultured with MSC primed or not (−) with CM_GM_ or CM_M_. Co-cultures were incubated in the absence or presence of neutralizing antibody (Ab) against IL-6 or PGE_2_. *p < 0.05

To examine whether TNF-α and IL-10 secretion from macrophages was regulated by PGE_2_ and IL-6, co-cultures were incubated with neutralizing antibodies against these mediators. TNF-α levels were similar in co-cultures incubated with anti-PGE_2_ and in macrophages cultured in isolation (Fig. 6a, b, left panels). In contrast, TNF-α levels in co-cultures treated with anti-IL-6 were lower than in isolated macrophages. The regulation in TNF-α levels induced by MSC primed with CM_GM_ or CM_M_ was suppressed by blocking PGE_2_ but not IL-6 (Fig. 6a, b, left panels). IL-10 levels in co-cultures were hardly affected by incubation with neutralizing antibodies. The only effect was observed in co-cultures of MΦ_GM_ as the increase in IL-10 levels induced by CM_GM_-primed MSC was attenuated when PGE_2_ was blocked (Fig. 6a, right panel). These data show that primed MSC co-cultured with macrophages decrease TNF-α levels through the secretion of PGE_2_. PGE_2_ is also involved in the regulation of IL-10 in co-cultures of primed MSC with MΦ_GM_ but not with MΦ_M_.

### Primed MSC encapsulated in collagen hydrogels promote macrophage anti-inflammatory versus pro-inflammatory cytokine secretion

**Fig. 7:**
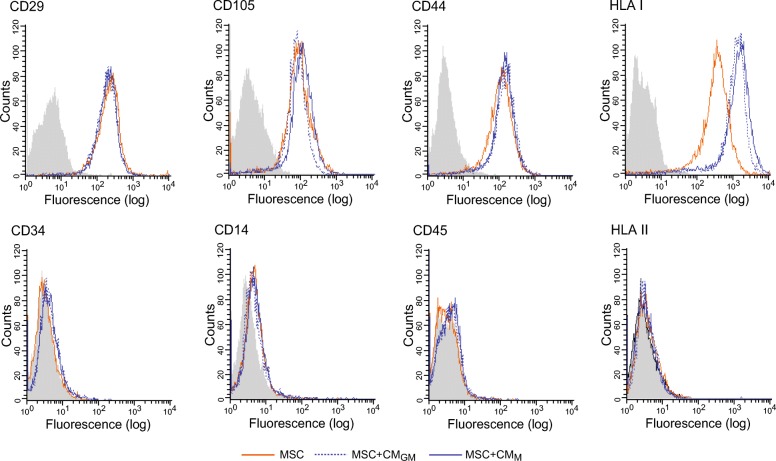
Effect of factors secreted by macrophages on MSC identity. Flow cytometric determinations of the expression of surface markers in MSC primed or not with CM_GM_ or CM_M_. Gray-filled histograms correspond to cells non incubated with antibodies

**Fig. 8:**
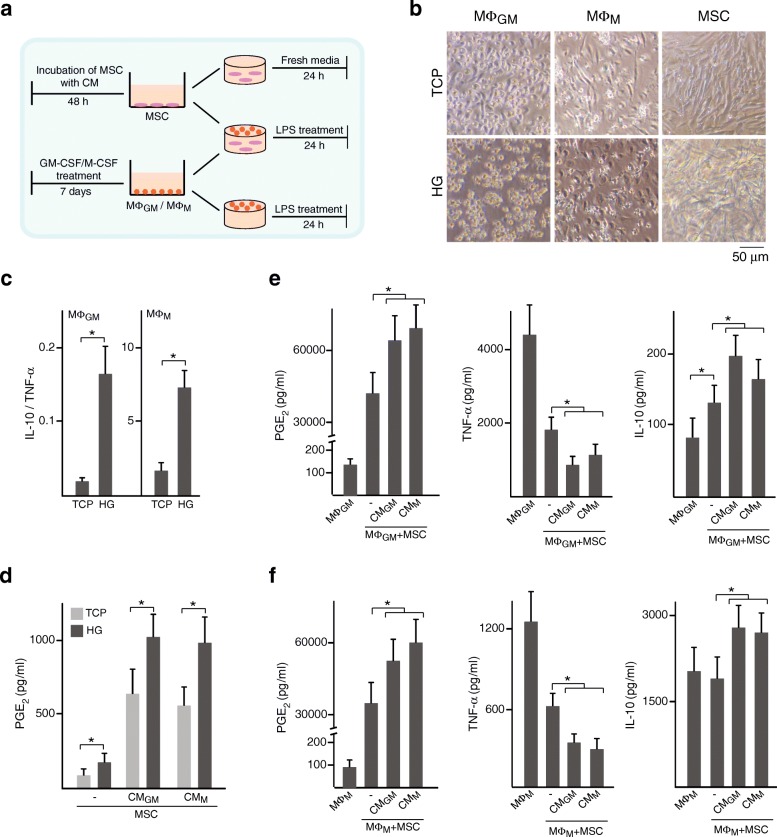
Regulation of macrophage cytokine secretion by MSC loaded in HG. **a** Scheme of MSC treatment with CM and cell culture in HG (upper row). Scheme of macrophage culture on HG loaded with MSC primed or not with CM (middle row) or cultured on HG lacking MSC (lower row). **b** Images of MΦ_GM_ or MΦ_M_ cultured on TCP or HG and of unprimed MSC cultured on TCP or encapsulated in HG. **c** Ratio between IL-10 and TNF-α levels in media of MΦ_GM_ or MΦ_M_ cultured on TCP or HG. **d** PGE_2_ levels in media of MSC primed or not with CM and cultured on TCP or encapsulated in HG. PGE_2_, TNF-α and IL-10 levels in media of MΦ_GM_ (**e**) or MΦ_M_ (**f**) cultured on empty HG or on HG loaded with MSC primed or not with CM. *p < 0.05

Immune rejection of allogeneic MSC has been associated with an alteration in HLA expression following exposure to inflammatory factors [20, 21]. Cell surface levels of HLA class I increased after treatment of MSC with CM, while no effect was observed for HLA class II (Fig. 7). Expression levels of other cell surface molecules related to MSC identity were not altered by CM treatment (Fig. 7). Encapsulation of MSC in HG has been proposed as a strategy to enhance their survival after transplantation and potentiate their function. We extended our study to explore the immunomodulatory properties of CM-primed MSC encapsulated in collagen HG, where cells are permitted to grow in three dimensions. To this end, MΦ_GM_ or MΦ_M_ were seeded on HG, loaded or not with MSC, and stimulated with LPS. Macrophages or MSC were also cultured on TCP, as classical 2D cell growth conditions (Fig. 8a). On TCP, most MΦ_GM_ acquired a polygonal morphology whereas the majority of MΦ_M_ exhibited an elongated spindle-like shape (Fig. 8b). However, both MΦ_GM_ and MΦ_M_ adopted a rounded shape when seeded on HG, being this morphological change more evident for MΦ_GM_. MSC encapsulated in HG were more spindle shaped than cells cultured on TCP (Fig. 8b). We found that the balance between IL-10 and TNF-α levels secreted from MΦ_GM_ or MΦ_M_ on HG suffered important alterations compared with TCP and was characterized by higher IL-10 to TNF-α ratio (Fig. 8c). PGE_2_ secretion from unprimed or primed MSC loaded in HG was higher than on TCP (Fig. 8d). As observed on TCP, priming with CM substantially increased PGE_2_ secretion from MSC in HG (Fig. 8d). This increase was also detected when MΦ_GM_ or MΦ_M_ were co-cultured on the HG surface (Fig. 8e, f, left panels). Incubation of MSC with CM before encapsulation in HG enhanced their immunomodulatory properties on macrophages. Thus, MΦ_GM_ or MΦ_M_ cultured on MSC-loaded HG secreted lower TNF-α levels than macrophages cultured on empty HG, an effect further enhanced when MSC were primed with CM (Fig. 8e, f, middle panels). Interestingly, IL-10 secretion from MΦ_GM_ increased when HG were loaded with MSC and further increased when MSC were primed with CM (Fig. 8e, right panels). High IL-10 levels secreted by MΦ_M_ on HG were unaffected by loading unprimed MSC whereas increased when MSC were primed with CM (Fig. 8f, right panels). In all cases, similar effects were observed by priming MSC with CM_GM_ or CM_M_. Taken together, these data indicate that MSC encapsulated in HG enhance the ratio of IL-10 to TNF-α levels secreted by MΦ_GM_ or MΦ_M_ and that these immunoregulatory effects are potentiated by priming MSC with factors secreted by macrophages.

## Discussion

Immunomodulatory effects of MSC are the result of an integrated response to extracellular stimuli, which change during the course of wound healing. MSC require threshold levels of inflammatory factors to activate their immunosuppressive function whereas insufficient MSC activation may contribute to potentiate inflammation [10, 12]. Based on these observations, treatment of MSC with inflammatory factors prior to implantation has emerged as an attractive strategy to boost their immunoregulatory effects, as shown in animal models of colitis, acute myocardial ischemia, GvHD, and tendon and ligament healing [22–26]. Classical pro-inflammatory cytokines released at the early stage of inflammation such as IFN-γ, TNF-α, or IL-1β potentiate paracrine effects of MSC on macrophages [23, 27–29]. In this work, we show for the first time that factors originated from pro-inflammatory or anti-inflammatory macrophages enhance immunomodulatory properties of MSC. Our data show that MSC immunomodulation was enhanced by priming MSC with CM from LPS-stimulated MΦ_GM_ or MΦ_M_ but not by CM from unstimulated macrophages, supporting the notion that MSC are mainly activated by inflammatory factors. Priming MSC with CM_GM_ promoted MΦ_GM_ polarization toward an anti-inflammatory phenotype, as evidenced by reduced TNF-α levels and increased IL-10 secretion. Blocking TNF-α in CM_GM_ significantly attenuated the immunomodulatory effects of primed MSC indicating that TNF-α acts as a priming factor for MSC and therefore plays a critical role in MSC and macrophage interactions. Interestingly, MSC primed with CM_M_ reduced TNF-α secretion from MΦ_GM_ to a similar extent than MSC primed with CM_GM_, suggesting that MSC can be activated by the cytokine milieu of damaged and repairing tissue. To our knowledge, it remains unclear whether anti-inflammatory factors influences MSC activation. Our data show for the first time that IL-10 originated from anti-inflammatory macrophages contributes to potentiate immunomodulatory functions of MSC. We found that the ability of primed MSC to reduce TNF-α secretion from MΦ_GM_ was enhanced by the content of TNF-α and IL-10 in CM_M_. IL-10 alone was insufficient to potentiate MSC immunomodulation, but enhanced the priming effect of TNF-α. These results indicate that MSC activation by IL-10 is dependent on TNF-α and suggest that IL-10 may act amplifying MSC activation by pro-inflammatory factors rather than as a priming factor. It is interesting to note that besides TNF-α and IL-10, CM contain a large range of soluble factors that may contribute to prime MSC. The optimal timing for MSC delivery remains uncertain and likely depends on the inflammatory environment associated with a specific disease or disorder. MSC administration at the early inflammatory stage, rather than after disease stabilization, seems to better guarantee the achievement of immunosuppressive activity in the case of acute GvHD [30]. However, the repair phase after acute myocardial infarction may be a more favorable time for MSC administration than during the acute injury phase, in which the hostile microenvironment could impair survival of transplanted cells [31]. Data herein suggest that MSC may be effective in modulating the immune response when transplanted at the onset of resolution, when both pro-inflammatory and anti-inflammatory factors are secreted [6], facilitating an effective transition from the pro-inflammatory phase to tissue repair.

MSC-mediated immunomodulation involves a complex network of cytokines as well as cell to cell interactions. PGE_2_ exert anti-inflammatory effects on macrophages via the cyclic AMP-responsive element (CRE) binding proteins (CREB), which regulate the transcription rates of several immune-related genes, including TNF-α and IL-10, upon binding to CRE present in their promoter regions [32]. More recently, it has been described that IL-6 regulates anti-inflammatory macrophage polarization although underlying mechanism has not been fully elucidated [33, 34]. Using blocking antibodies, we demonstrated that IL-6 and PGE_2_ mediate the reduction in TNF-α secretion from MΦ_GM_ in co-cultures with MSC. Interestingly, CM_GM_ and CM_M_, which contained high and low levels of pro-inflammatory factors, respectively, were similarly effective in stimulating PGE_2_ secretion by MSC. This effect may be explained by the IL-10-induced increase in PGE_2_ levels in the presence of low or high concentrations of TNF-α. In this regard, IL-10 has been shown to enhance MSC activation by other inflammatory factors, as production of IFN-β and IL-10 by regulatory T cells synergistically induced expression of the immunoregulatory factor indoleamine 2,3-dioxygenase (IDO) at the mRNA level in MSC [35]. However, we did not detect IDO protein levels in the media of the various cultures and co-cultures of unprimed or primed MSC (data not shown). Changes in PGE_2_ secretion in co-cultures paralleled changes in *COX2* mRNA levels in MSC indicating that production of this mediator was regulated at the mRNA level. The ability of primed MSC to further decrease TNF-α secretion by MΦ_GM_ could be attributed to PGE_2_ but not to IL-6, as indicated in the experiments using neutralizing antibodies against these mediators. These data support the notion that MSC immunomodulatory potential is strongly related to the production of PGE_2_ and suggest that enhancement of the production of this immunoregulatory factor by anti-inflammatory stimuli occurs at the onset of resolution.

It is interesting to note that co-culturing MΦ_GM_ with unprimed MSC or with MSC primed with CM_M_, which contained low levels of pro-inflammatory factors, failed to increase IL-10 secretion. MSC may require strong activation in a pro-inflammatory milieu to promote anti-inflammatory signatures in MΦ_GM_ as MSC primed with CM_GM_ led to increased IL-10 levels in co-cultures. Recent in vitro and in vivo studies show that priming MSC with TNF-α at 10 ng/ml or higher doses favors macrophage polarization toward an anti-inflammatory phenotype [22, 29]. In fact, we detected that blocking TNF-α in CM_GM_ reduced the ability of primed MSC to increase IL-10 secretion from MΦ_GM_ and that priming MSC with 10 ng/ml TNF-α alone enhanced IL-10 levels in co-cultures. Reprograming macrophages to an anti-inflammatory phenotype has been shown to be mediated by PGE_2_ [36]. Supporting this, neutralization of PGE_2_ in co-cultures of MΦ_GM_ and MSC primed with CM_GM_ reduced IL-10 levels. We speculate that PGE_2_ may promote IL-10 secretion from MΦ_GM_ via the CREB signaling pathway, as described in cultures of mouse bone marrow macrophages [37]. Notably, priming MSC with CM_M_, which increased PGE_2_ secretion, did not result in increased IL-10 production in co-cultures with MΦ_GM_, indicating that other factors secreted by MSC cooperate with PGE_2_ in macrophage phenotype switching.

Paracrine interactions between MSC and anti-inflammatory macrophages have been scarcely investigated. MSC, primed or not with CM from macrophages, regulated TNF-α secretion from MΦ_M_ in a similar way to that observed in co-cultures with MΦ_GM_ whereas IL-10 production showed different trends. As observed by others [38], IL-10 levels secreted by MΦ_M_ decreased after co-culturing with unprimed MSC. In contrast, MΦ_M_ maintained their anti-inflammatory traits when co-cultured with MSC primed with inflammatory factors. These results suggest that the cytokine environment strongly influences the ability of MSC to control anti-inflammatory functions of macrophages, allowing resolution of inflammation or preventing excessive anti-inflammatory activation that could impair tissue healing. Moreover, paracrine effects of MSC on MΦ_M_ could be regulated by anti-inflammatory factors secreted in the resolution of inflammation, as suggested by the data from experiments in which MSC were primed with a high concentration of IL-10. It should be mentioned that changes in IL-10 levels in co-cultures of MΦ_M_ were independent of the PGE_2_ content, suggesting that different signaling pathways regulate IL-10 production in pro-inflammatory and anti-inflammatory phenotypes.

Murine MSC upregulated the expression of MHC class II molecules in response to IFN-γ and were rejected after implantation in immunocompetent MHC-mismatched mice [39–41]. In human MSC, the expression of both HLA classes I and II increased after treatment with IFN-γ [20]. Our data show that treatment of MSC with CM increased surface expression of HLA class I but not of HLA class II, which could be attributed to an inhibitory effect of factors contained in the CM. For example, transforming growth factor-β (TGF-β) has been shown to reduce IFN-γ-induced expression of HLA class II in human MSC [42]. One approach to improve stem cell-based therapies is the use of biomaterial carriers. Type I collagen HG have been successfully used as drug delivery vehicles for the treatment of long bone fracture and spinal fusion [43]. Moreover, collagen HG have been explored to increase MSC survival after implantation and prevent immune rejection in vivo [44]. We observed that collagen HG are instructive microenvironments for macrophages and MSC functions as well as for the crosstalk between both cell types. Collagen HG substantially decreased pro-inflammatory activation of MΦ_GM_ and potentiated anti-inflammatory activity of MΦ_M_ as compared to 2D substrates. Increased PGE_2_ secretion and greater immunomodulatory activity were observed in MSC cultured in three-dimensional (3D) topographies [45] or in spheroids [46], effects that were attributed to 3D disposition of MSC. Our data suggest that encapsulation of MSC in HG is an effective approach to enhance immunomodulatory properties of MSC. MSC in HG promoted anti-inflammatory switching of MΦ_GM_, as indicated by marked reduction in TNF-α levels and increase in IL-10 production, and sustained MΦ_M_ activation characterized by high IL-10 secretion. Priming with CM conferred to MSC loaded in HG greater immunomodulatory potential, promoting MΦ_GM_ polarization toward an anti-inflammatory phenotype and supporting MΦ_M_ anti-inflammatory activation. Enhanced immunoregulatory effects of primed MSC in HG were probably a result of the substantial increase in PGE_2_ levels compared to unprimed counterparts. Taken together, our results show that priming MSC with inflammatory factors originated from macrophages enhances their immunomodulatory potential. In fact, recent preclinical data supports the safety of IFN-γ-primed MSC infused in mice and their effectiveness to treat immune-related diseases [23, 47]. Encapsulation of primed MSC in HG could be an effective approach to improve their therapeutic efficacy upon implantation. Further studies are required to elucidate the in vivo immunomodulatory potential of primed MSC loaded in HG.

## Conclusions

Factors secreted by pro-inflammatory and anti-inflammatory macrophages activate the immunomodulatory potential of MSC. This was attributed, at least in part, to the priming effect of TNF-α and was associated with an increase in PGE_2_ production by MSC. We identified that IL-10 secreted from anti-inflammatory macrophages, in combination with other inflammatory factors, activate MSC to secrete PGE_2_ and potentiate the priming effect of TNF-α. Encapsulation of primed MSC in collagen HG enhances their immunoregulatory function, promoting anti-inflammatory activity of macrophages. These findings contribute to the understanding of the mechanisms by which macrophages polarization dynamics instruct MSC and may provide a basis for the development of novel strategies to enhance MSC immunoregulatory potential.

## Additional file

Additional file 1: **Figure S1.** Flow cytometric determinations of the expression of surface markers in MΦ_GM_ or MΦ_M_. Gray-filled histograms correspond to cells non incubated with antibodies. **Figure S2.** Immunomodulatory effects of primed MSC co-cultured with macrophages in the absence of LPS. (a) Scheme of the set-up of co-cultures. MΦ_GM_ or MΦ_M_ were treated with LPS, washed with PBS, and then co-cultured in fresh media with MSC primed with CM from macrophages. TNF-α and IL-10 levels in media of co-cultures of MΦ_GM_ (b) or MΦ_M_ (c). *p < 0.05. (PDF 403 kb)
