# Supplementary material for: Immunoregulatory potential of mesenchymal stem cells following activation by macrophage-derived soluble factors
Source: Stem Cell Res Ther. 2019 Feb 13;10:58. doi: 10.1186/s13287-019-1156-6 (PMC6375172; doi:10.1186/s13287-019-1156-6)
Supplement: Supplementary file 1 — Figure S1. Flow cytometric determinations of the expression of surface markers in MΦGM or MΦM. Gray-filled histograms correspond to cells non incubated with antibodies. Figure S2. Immunomodulatory effects of primed MSC co-cultured with macrophages in the absence of LPS. (a) Scheme of the set-up of co-cultures. MΦGM or MΦM were treated with LPS, washed with PBS, and then co-cultured in fresh media with MSC primed with CM from macrophages. TNF-α and IL-10 levels in media of co-cultures of MΦGM (b) or MΦM (c). *p < 0.05. (PDF 403 kb) [file 13287_2019_1156_MOESM1_ESM.pdf]

Additional File 1

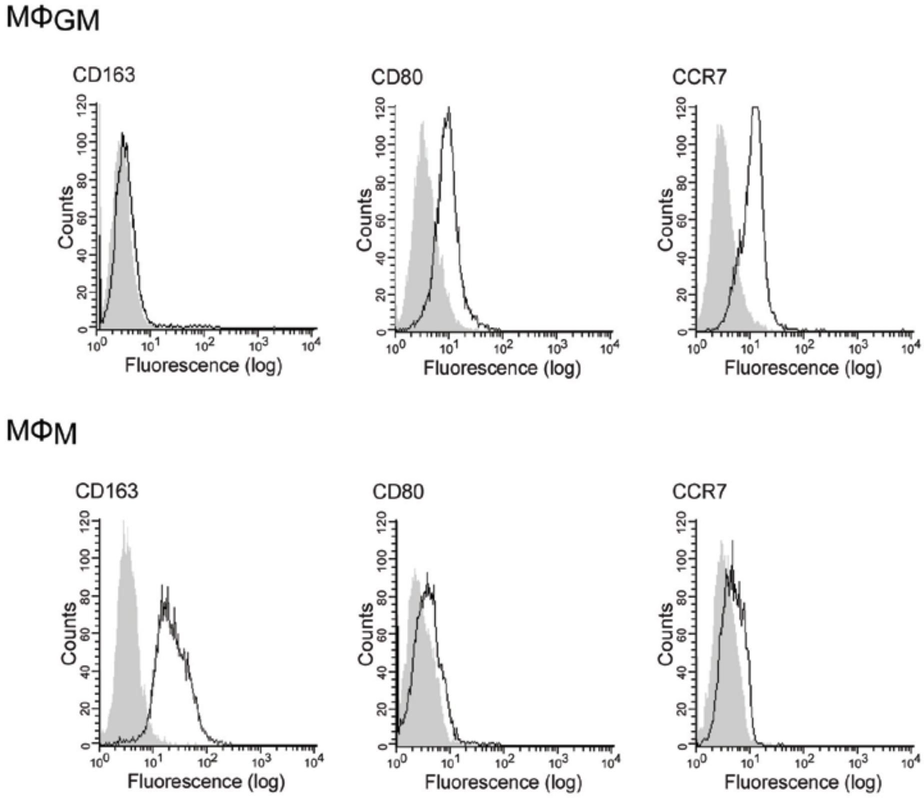

Figure S1. Flow cytometric determinations of the expression of surface markers in $M\Phi_{GM}$ or $M\Phi_{M}$. Gray filled histograms correspond to cells non incubated with antibodies.

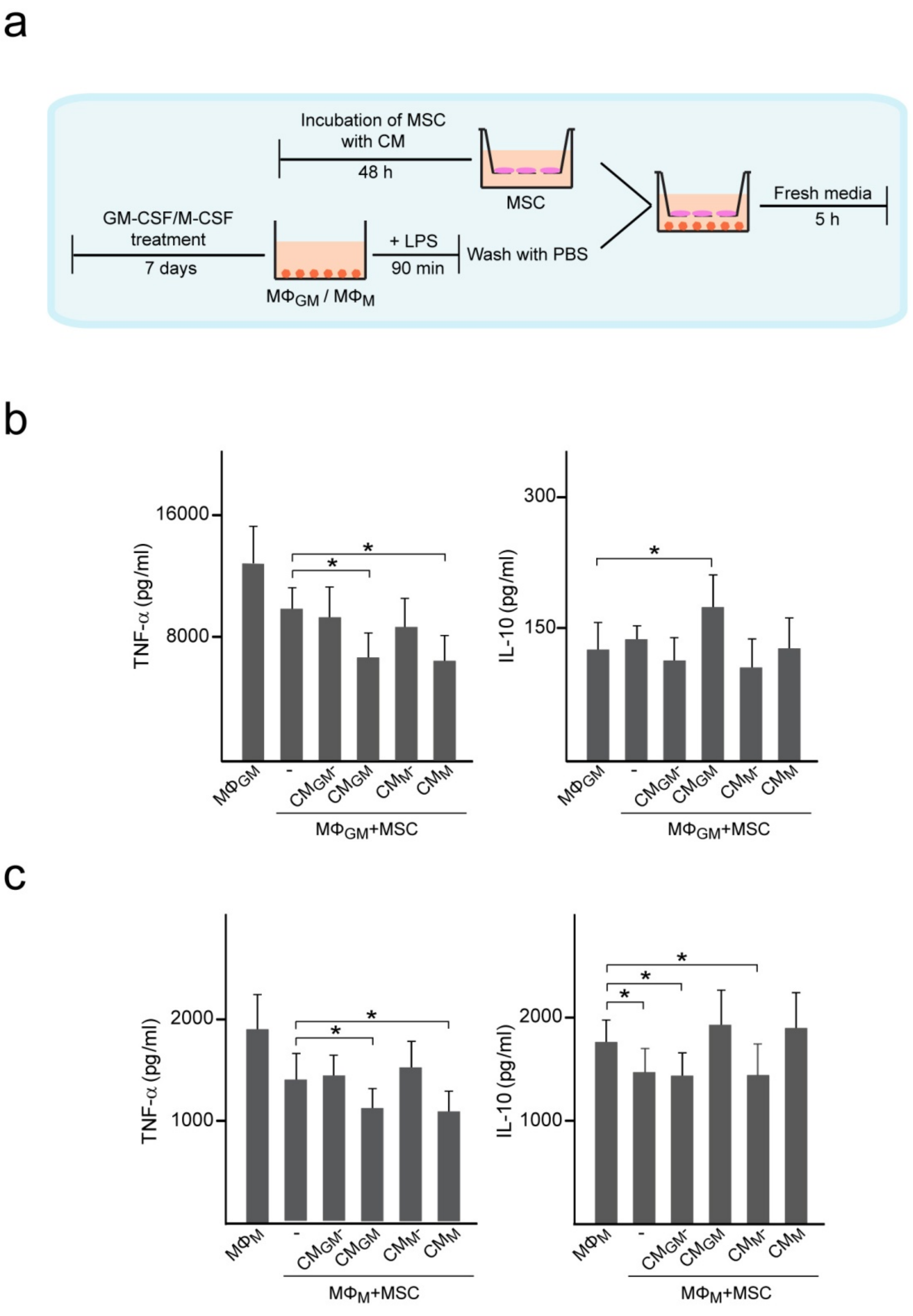

Figure S2. Immunomodulatory effects of primed MSC co-cultured with macrophages in the absence of LPS. (a) Scheme of the set-up of co-cultures. $M\Phi_{GM}$ or $M\Phi_{M}$ were treated with LPS, washed with PBS, and then co-cultured in fresh media with MSC primed with CM from macrophages. TNF-α and IL-10 levels in media of co-cultures of $M\Phi_{GM}$ (b) or $M\Phi_{M}$ (c). $^{*}p < 0.05$.
